# Environmentally sustainable practices in global health research and higher education institutions: Lessons from consultation with the TropEd Global Health institutions

**DOI:** 10.1111/tmi.13714

**Published:** 2022-01-10

**Authors:** Kate Whitfield, Alexandru Cretu, Teun Bousema, Justin Cohen

**Affiliations:** ^1^ Barcelona Institute for Global Health ISGlobal Barcelona Spain; ^2^ Department of Medical Microbiology & Radboud Center for Infectious Diseases Radboud University Medical Center Nijmegen The Netherlands; ^3^ Department of Infection Biology, Faculty of Infectious and Tropical Diseases London School of Hygiene & Tropical Medicine London UK; ^4^ Clinton Health Access Initiative Boston Massachusetts USA

**Keywords:** climate change, climate justice, global health, sustainability

## Abstract

**Objective:**

To examine how global health institutions are reducing the greenhouse gas emissions from their own operations and analyse the facilitators and barriers to achieving decarbonisation goals.

**Methods:**

We reviewed the sustainability goals and implementation plans of 10 global health universities from the ʻTropEdʼ network. We systematically collected information from institutional websites and annual reports. Through online interviews, 11 key informants validated the information from 9 of the institutions and shared their opinions regarding what factors are helping their institutions decarbonise and what factors are hindering progress.

**Results:**

4/10 institutions sampled have a sustainability strategy and implementation plan, only 3/10 have specific decarbonisation goals, and 3/10 are reporting on progress. 5/10 institutions reported that they are in the process of determining emission reduction targets.

**Conclusion:**

This paper identifies common success factors that facilitate decarbonisation as well as common challenges and how they are being tackled, and makes recommendations on sustainability efforts in academic institutions.

## INTRODUCTION

Globally, climate change is the greatest threat to health, well‐being and livelihoods [1, 2, 3]. The average surface temperature of the planet is now approximately 1.2°C higher than in pre‐industrial times, and the associated health impacts are evident [2, 4]. Vulnerable groups such as pregnant women, children, individuals with concomitant conditions, the elderly and people living in low‐income settings are disproportionately affected [2]. Governments, civil society and all sectors are grappling with the need to rapidly decarbonise to prevent global temperatures from rising above 1.5°C and to prevent the worst effects of climate change as agreed in the Paris Climate Accord [5].

Global health research institutions have a pivotal role to play in the achievement of the Sustainable Development Goals (SDGs) and the Paris Climate Accord since their mission is ultimately to improve the health and well‐being of populations, particularly the most vulnerable [5, 6, 7, 8]. Like any large enterprise, the operations of global health research institutions create carbon emissions and have an impact on the environment [9]. It is incumbent upon the global health community to reduce its own greenhouse gas emissions in line with the latest Intergovernmental Panel on Climate Change (IPCC) guidance.

This paper reports on how ten European global health research institutions in the ‘TropEd’ network for education in international health are transitioning to environmentally sustainable operations and reducing their carbon footprint [10]. The TropEd institutions conduct research, education and field work in global health. We spoke to 11 key informants from nine of the 10 member institutions in the network to ascertain the common success factors in implementing environmentally sustainable operations and how to tackle the challenges.

## METHODS

### Understanding sustainability efforts

We examined the 10 ‘home institutions’ in the TropEd network for education in international health [10]. These global health research institutions aim to improve global health. Given the negative effect of greenhouse gas emissions on global health, it is relevant to learn about the extent to which they are shrinking their own carbon footprints and what the facilitators and barriers to progress are so that other institutions can learn from their lessons.

We considered sustainability as the process of designing and implementing strategies to reduce and measure greenhouse gas emissions and other negative environmental impacts generated by the institution's operations whilst advancing the core mission of the institution. Between February and March 2021, we systematically searched the institution's websites and latest annual reports for information about sustainability initiatives to collect information about goals to reduce the carbon footprint, action plans and progress. We used a data collection framework as detailed in Table 1.

**TABLE 1:** Data collection framework

General information	Sustainability strategy	Sustainability actors	Details of sustainable initiative	Facilitators and barriers
Name of the organisation Relationship with the university Country Website	Core value/guiding principle Institutional policy/statement Sustainability goals Implementation plan	Decision‐makers Dedicated role/office Student body Volunteer groups	Goals to reduce carbon emissions Measuring carbon emissions Action in thematic areas · Energy · Transport · Water · Waste and recycling Education and training Reporting	Facilitators Barriers

Note

We used this framework to collect data on environmentally sustainable practices in the 10 TropEd member institutions and to structure the key informant interviews.

Between March and August 2021, we conducted 60‐min virtual interviews with key informants from each TropEd institution. We searched the institution websites for contact persons relating to sustainable operations, research or education in sustainability and reached out to them via email. We also used general information or department email addresses to ask to be put in touch with the appropriate person. If we were unable to find a contact person, we worked with the coordination team of the TropEd network to reach out to member institutions. If there was more than one key informant, we conducted a small group interview, and if anyone was not comfortable taking part in an interview in English, we offered to do the interview with a translator. With permission, we recorded the interviews and transcribed the audio using the Sonix online tool to help take note of all the information [11].

Before the interviews, we shared the information that we had collected online using the data collection framework (Table 1) with the key informants. The first step of the interviews was to check that what we had collected from the online sources was correct and complete. If the key informant provided additional information that was not publicly available, we noted it separately in the data collection. We also asked each key informant their opinion regarding factors that were facilitating progress and factors that were acting as barriers to progress. The interviews were the main source of information on success factors and challenges. We present the aggregated results.

## RESULTS

### Implementation of sustainability efforts in 10 global health research institutions: progress; common success factors and challenges

We collected information from all TropEd institutions and conducted key informant interviews with 11 representatives of 9 out of ten TropEd institutions (Table 2), and we were unable to conduct an interview with staff from the Institute for Global Health, University College London. Online information was not available in English from three of the TropEd institutions. The 10 home institutions are located across Europe, some are independent, and some are part of a university.

**TABLE 2:** Overview of the 10 TropEd member institutions

Global health research institution	Country	Website and documents examined
Institute of Tropical Medicine, Antwerp (ITM)	Belgium	https://www.itg.be Annual reports 2018 and 2019
Center for International Health, Ludwig‐Maximilian‐University Munich (LMU)	Germany	https://www.cih.lmu.de LMU sustainability policy v1.0 and LMU sustainable development report 2019
Institute of Global Health, University of Heidelberg (UH)	Germany	https://www.klinikum.uni‐heidelberg.de/heidelberger‐institut‐fuer‐global‐health/ UH Annual report 2018 and 2019 (German)
Institute of Tropical Medicine and International Health of Charité, University of Medicine, Berlin (CB)	Germany	https://tropeninstitut.charite.de/en Charité Berlin annual report 2019, Charité Berlin strategy 2030 (German)
Clinic of Infectious and Tropical Diseases, University of Brescia (UNIBS)	Italy	https://en.unibs.it/ UNIBS waste management regulation 2014 (Italian), UNIBS Sustainability report 2017–19 (Italian), UNIBS sustainable development plan 2018 (Italian), UNIBS strategic plan 2017–19 and 2020–22 (Italian), UNIBS Statute 2020
KIT Royal Tropical Institute (KIT)	The Netherlands	https://www.kit.nl KIT annual reports 2017, 2018 and 2019, KIT green office policy document 2016, Knowledge for a sustainable world at KIT, KIT sustainability manual 2020, Leaflet on sustainability at KIT 2019
Institute for Global Health Barcelona (ISGlobal)	Spain	https://www.isglobal.org/en ISGlobal report academic year 2018–19, Statute ISGlobal (Spanish), ISGlobal strategic plan 2019–23, ISGlobal annual report 2019
Swiss Tropical and Public Health Institute (Swiss TPH)	Switzerland	https://www.swisstph.ch Swiss TPH annual report 2019
Institute for Global Health and Development, Queen Margaret University (QMU)	UK	https://www.qmu.ac.uk/schools‐and‐divisions/ighd QMU strategic plan 2020–25, QMU travel survey report 2018–19, QMU green travel plan 2020, QMU climate change action plan 2017–22
Institute for Global Health, University College London (UCL)	UK	https://www.ucl.ac.uk/global‐health UCL greening the recovery, Change possible: strategy for a sustainable UCL 2017–24, Sustainable UCL annual report 2018–19

Note

We collected publicly available information from all 10 institutions and conducted key informant interviews with individuals from all institutions to validate the information and to fill in any gaps, except the Institute for Global Health, University College London (UCL).

The information available online was very heterogeneous in terms of content and level of detail. The key informants corroborated the information we collected from online sources and added further information. The key informants had a range of positions in their institutions, including academic faculty and administrative staff. They all identified themselves as being engaged in topics that were directly related to sustainable operations in their workplace, be that through a voluntary role in a committee, or an explicit part of their job description.

**FIGURE 1:**
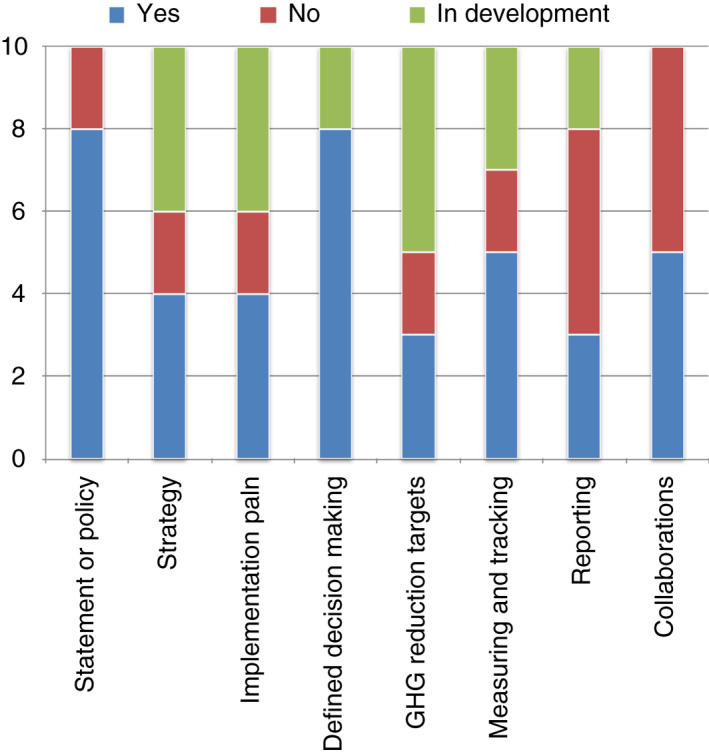
Chart of sustainability efforts in the 10 TropEd institutions. The number of global health research institutions from the 10 TropEd member institutions with components relating to environmental sustainability. The various components were either ‘yes’ already in place, ‘no’ not in place or ‘in development’

All of the Troped institutions had embraced the United Nations (UN) SDGs and 2030 agenda as a strategic framework [6]. The SDGs are considered a cross‐cutting issue relevant to their core business of research and education as well as operations. Of the ten global health research institutions, 8 have a written statement or policy regarding sustainability, 4 have a strategy and implementation plans in place, and 4 are developing implementation plans. Only 3 have specific targets to reduce their carbon footprint; however, another 5 institutions are developing targets. See Figure 1. This information was validated for all institutions, except the Institute for Global Health, University College London.

### Common success factors and challenges

Here, we present the common factors that are facilitating progress in sustainable operations across the global health research institutions, as well as common barriers and how the global health research institutions are tackling them. See Table 3. In the section below, we describe some themes that emerged from the key informant interviews.

**TABLE 3:** Common factors that facilitate progress, common challenges and ways to overcome them

**Facilitators**
UN SDG agenda as a strategic framework
Leadership from the directorate plus inclusive engagement from all internal stakeholder groups
Pressure and demand from internal stakeholders, especially the student body by inquiring, acting and demanding action
Clearly defined priorities for action that avert the greatest amount of greenhouse gas emissions
Clear roles and responsibilities for sustainable operations
Local and national legislation that promote sustainable practices and engagement with local community actors
**Common challenges and solutions being applied**
Significant emissions from air travel · Increase participation in virtual meetings · Prioritise travel by train · Open a discussion and scrutinise which trips by air are really essential · Offset greenhouse gas emissions for essential air travel
Poor energy efficiency of historic and protected buildings · Subsidies for retrofitting · Reduce heating based on gas and choose a provider that offsets emissions · Electrify heating systems, choose an electricity provider that is expanding the amount of renewable energy that feeds into the grid or install renewable energy systems · Switch to heating systems that do not use gas such as biomass boilers and heat pumps
Resistance to change and adopting more sustainable ways of working · Engage all stakeholders in awareness‐raising and practical solutions · Share best practices · Incentivise behaviour change (e.g. foster friendly competition, awards and reduced prices on public transport)
Uncertain budgets that impede long‐term planning · Cost‐benefit analysis · Investments framed as part of the SDG long‐term agenda

#### Being inclusive and setting priorities

Many key informants cited engagement of all internal stakeholder groups (e.g. staff, researchers and students) as a key success factor. Insights, ideas and actions from a diverse cross‐section of an institution generated diverse solutions. Key informants reported that when sustainability was embedded into everyone's roles and responsibilities through the organisation, it becomes a part of everyone's day‐to‐day experience and part of the working culture.

Key informants also flagged the importance of prioritising sustainability activities to those that will be most impactful. Sustainability is a broad agenda, and different stakeholders have different concerns. Informants spoke to the importance of prioritising resources and action where the greatest carbon savings could be made. In one example, carbon footprint analysis showed that the greatest sources of emissions were from air travel. This is unsurprising given the global nature of global health. One key informant suggested that the number of in‐person meetings of the Trop Ed network itself should be reduced and locations chosen based on accessibility by train.

#### Working with partners

Action to tackle climate change is of increasing importance to the general public and more policy‐makers, public and private sector stakeholders are leading change. A number of key informants reported that engagement with local actors was an effective way to galvanise action and help create a shift towards a lower carbon working culture. A notable example from the University of Brescia was the signing of an accord between local actors at the top of a mountain where the glacier was melting.

One key informant highlighted the importance of partnerships between different global health research institutions, with many partnerships existing between global health institutions in high‐income countries (HICs) and those in endemic settings, which are often low‐ and middle‐income countries (LMICs). The key informant made the point that these research and training partnerships should also include working together on sustainability efforts.

All key informants agreed that global‐level collaboration was also of value; however, none were participating in consortia such as the United Nations Environment Programme Green Nudges initiative, or Race to Zero, My Green Lab or laboratory efficiency assessment framework (LEAF) [12, 13, 14, 15].

#### Funding

Several key informants pointed to funders as a barrier to progress. Short‐term funding and uncertain budgets impeded larger‐scale investments. Largely, funders do not incentivise low carbon travel or environmentally sustainable practices by grantees. In one example, old electronics were being destroyed to demonstrate compliance with funder requirements that items could not be repaired and re‐sold.

However, it was local, regional and national government schemes that were offering programmes to subsidise investments in improving the energy efficiency of old and historic buildings in some instances.

#### Lessons from the COVID pandemic

The key informants discussed the impact of the pandemic on their institutional sustainability agendas. A digital shift has taken place during the course of the pandemic. The academic community is sharing research findings, coordinating projects, conducting interviews and learning online. This has resulted in a massive reduction in greenhouse gas emissions from air travel and commuting by car, but this gain for the climate has come at a devastating cost for human health and well‐being. It is therefore incumbent upon us to maintain these new low carbon ways of working and not waste this lesson that came at such an enormous cost [16].

## DISCUSSION

Reducing the carbon footprint of global health research institution activities aligns with organisational purpose to improve global health. We found that different institutions are in different stages of developing and implementing a sustainability agenda, and this is a rapidly evolving area. For the purposes of this work, we focused on sustainability in terms of greenhouse gas emissions as the main driver of climate change. Here, we offer our thoughts as to how institutions in global health research and higher education can strengthen their sustainability strategies and implementation plans with a specific focus on reducing emissions. These principles can apply to any field, not just global health.

At the core of an institutions' sustainability strategy needs to be an overarching goal to reduce greenhouse gas emissions in line with the most recent climate science [17]. Emissions that are generated from all work or study‐related activities need to be included in the reduction strategy, and the baseline and annual emissions should be reported internally and publicly. The Greenhouse Gas Protocol (GHG Protocol) is a globally recognised set of tools to measure and track emissions [18]. The GHG Protocol categorises emissions into three scopes: scope 1 emissions are generated directly from sources owned or controlled by the organisation; scope 2 emissions are caused indirectly from the generation of purchased energy; and scope 3 emissions are caused indirectly are a result of the organisation's operations and activities [19]. Due to the nature of research and higher education, most emissions generated in academic institutions are scope 2 from the consumption of electricity and gas to power offices, laboratories, data centres, teaching facilities and residencies and scope 3 from commuting, air travel, food, all purchasing and waste [18]. The GHG Protocol also offers online training on how to estimate emissions [20]. In addition, carbon emissions calculations and methods are well described in a recent paper from a group of astronomy research institutions in the Netherlands and an online tool to calculate emissions from air travel is available from the Institute de Recherche Astrophysique et Planetologie (IRAP) [21, 22, 23].

The Higher Education Climate Action Toolkit is a practical guide to devise and implement climate action plans [24]. The UN‐backed ‘Race to Zero’ offers a platform for universities and colleges to rapidly reduce emissions to ‘net zero’ [13]. The principles apply to any academic institution and are to set a goal; set near‐term targets; take action to reduce emissions; and measure and publicly report on emissions. The Race to Zero website hosts case studies and other resources, including guidance on how to ‘Get Net Zero Right’ [25].

Data centres and laboratories are the two most energy‐intensive settings per square metre, and My Green Lab and LEAF are two platforms for teams working in research (particularly in wet laboratories) to track and reduce the environmental impact of their work [14, 15].

Global health research institutions may have holdings or endowments invested in portfolios that include fossil fuel assets. Advocates have successfully called for universities to divest from oil and gas holdings over recent years [26, 27, 28]. Divestment would align endowment portfolios with the health goals of global health research institutions and demonstrate institutional commitments to sustainability. The London School of Hygiene and Tropical Medicine is among those institutions divesting their assets from fossil fuel investments and reporting on this transparently [29].

One concept that is very pertinent for institutions working in global health and education is climate justice [30, 31]. Given that the negative impacts of climate change disproportionately affects vulnerable groups, those living in low‐income settings and young people, institutions working in global health and education should integrate the principles of climate justice into the organisational sustainability agenda. For example, global health research institutions in HICs with close working partnerships with counterparts in LMICs should make efforts to be a partner on sustainable approaches. One example of this is the solar power installation at the MRC Unit The Gambia at the London School of Hygiene and Tropical Medicine [29]. Institutions with education missions should include training on skills suitable for the green economy into internal training programmes and outreach activities [32].

In many instances, action to reduce emissions requires that we change our behaviour, for example using active transport or public transport to get to work, choosing to connect to meetings online rather than flying and choosing plant‐based food and beverages. The United Nations Environment Programme (UNEP) Little Book of Green Nudges offers guidance on how to make the sustainable options easy, attractive, social and timely [12]. Institutions can also take part in the initiative by testing ‘green nudges’ and sharing lessons learned.

The TropEd members reported that engaging all staff, researchers and students in the institution's sustainability strategy was an important success factor. One way to engage and empower internal stakeholders in sustainability action is to offer training. The Carbon Literacy Project is a source of accredited training where participants develop their own sustainability actions to implement in their workplace or study setting [33].

Here, we set out a series of recommendations on how to integrate sustainable operations into global health research institutions (Box 1). They are pertinent to any academic or higher education institution in any field.

Box 1 **Recommendations**

Engaging people

1. Engage, build competencies and empower all internal stakeholders to participate and add value to sustainable operations in the workplace.
2. Clearly assign sustainability responsibilities for all internal stakeholders, in all job descriptions and terms of reference.

Working with collaborators

3. Collaborate with local partners to amplify impact and global partners to share lessons learned and maintain momentum. UNEP Green Nudges, Race to Zero, My Green Lab and LEAF are relevant for academic institutions.
4. Partner on sustainability with global health research institutions in LMIC and incorporate skills for the green economy into training, internship and youth outreach programmes. This helps build climate justice into the organisational sustainability agenda.
5. Discuss with funders with the aim of partnering to accelerate and strengthen the shift to sustainable practices.

Taking concrete and rapid action

6. Analysis and planning should not delay action to reduce emissions from the major sources such as energy use, transport and food.
7. Assess the institution's carbon footprint to prioritise actions that avert the most carbon emissions and to set carbon reduction targets.
8. Collect data overtime to ensure absolute reductions in emissions.
9. Report on progress internally and externally, including in the institutional annual report.

Some research funders are beginners to change their policies in support of greener operations, but far bolder and broader leadership from funders to incentivise and require sustainable operations in grantee institutions is needed. [34, 35, 36]. Stakeholders and grantees in academic institutions should be talking with their funders about how to work together to bring about sector‐wide change.

We recognise that taking these steps to measuring and reducing global health research institutions' emissions will require meaningful investment. In many cases, institutions will need to create new systems for measurement and enable a shift to a more sustainable working culture (e.g. embracing virtual meetings over frequent travel). Yet this evolution should be considered as a core pillar of building a strong, effective global health programme that does not contribute to climate change and poorer global health.
